# Multichannel Electrocardiograms Obtained by a Smartwatch for the Diagnosis of ST-Segment Changes

**DOI:** 10.1001/jamacardio.2020.3994

**Published:** 2020-08-31

**Authors:** Carmen Anna Maria Spaccarotella, Alberto Polimeni, Serena Migliarino, Elisa Principe, Antonio Curcio, Annalisa Mongiardo, Sabato Sorrentino, Salvatore De Rosa, Ciro Indolfi

**Affiliations:** 1 Division of Cardiology, Magna Graecia University, Catanzaro, Italy; 2 Center for Cardiovascular Research, Magna Graecia University, Catanzaro, Italy; 3 Mediterranea Cardiocentro, Naples, Italy

## Abstract

**Importance:**

Acute coronary syndromes are the leading cause of death worldwide and the leading cause of disease burden in high-income countries. Quick and accurate diagnosis of acute coronary syndromes is essential to avoid fatal events, for timely intervention, and to improve the prognosis.

**Objective:**

To prospectively investigate the feasibility and accuracy of a smartwatch in recording multiple electrocardiographic (ECG) leads and detecting ST-segment changes associated with acute coronary syndromes compared with a standard 12-lead ECG.

**Design, Setting, and Participants:**

A commercially available smartwatch was used in 100 participants to obtain multiple-channel ECGs. The study was conducted from April 19, 2019, to January 23, 2020. Fifty-four patients with ST elevation myocardial infarction, 27 patients with non-ST elevation myocardial infarction, and 19 healthy individuals were included in the study. The watch was placed in different body positions to obtain 9 bipolar ECG tracings (corresponding to Einthoven leads I, II, and III and precordial leads V1-V6) that were compared with a simultaneous standard 12-lead ECG.

**Main Outcomes and Measures:**

The concordance among the results of the smartwatch and standard ECG recordings was assessed using the Cohen κ coefficient and Bland-Altman analysis.

**Results:**

Of the 100 participants in the study, 67 were men (67%); mean (SD) age was 61 (16) years. Agreement was found between the smartwatch and standard ECG for the identification of a normal ECG (Cohen κ coefficient, 0.90; 95% CI, 0.78-1.00), ST-segment elevation changes (Cohen κ coefficient, 0.88; 95% CI, 0.78-0.97), and non–ST-segment elevation changes (Cohen κ coefficient, 0.85; 95% CI, 0.74-0.96). In addition, the Bland-Altman analysis demonstrated agreement between the smartwatch and standard ECG to detect the amplitude of ST-segment changes (bias, −0.003; SD, 0.18; lower limit, −0.36; and upper limit, 0.36). Use of the smartwatch ECG for the diagnosis of normal ECG showed a sensitivity of 84% (95% CI, 60%-97%) and specificity of 100% (95% CI, 95%-100%); for ST elevation, sensitivity was 93% (95% CI, 82%-99%) and specificity was 95% (95% CI, 85%-99%); and for NSTE ECG alterations, sensitivity was 94% (95% CI, 81%-99%) and specificity was 92% (95% CI, 83%-97%).

**Conclusions and Relevance:**

The findings of this study suggest agreement between the multichannel smartwatch ECG and standard ECG for the identification of ST-segment changes in patients with acute coronary syndromes.

## Key Points

**Question:**

Can a smartwatch record multiple-lead electrocardiograms (ECGs) and detect changes in the ST segment?

**Findings:**

In this case series including 100 participants, a smartwatch was able to record multichannel ECGs (leads I, II, III, V1, V2, V3, V4, V5, and V6) in agreement with standard ECGs. In addition, the amplitude of ST-segment changes noted with the smartwatch was comparable to those of standard ECGs.

**Meaning:**

The findings of this study suggest that use of ECGs recorded on smartwatches might be useful to obtain an earlier diagnosis of acute coronary syndromes; these data need to be further examined in patients with suspected myocardial infarction in whom false-positive and false-negative findings could be better characterized.

## Teaser

This case series evaluates the use of a smartwatch vs the standard measure of electrocardiographic recordings in patients with acute coronary syndromes.

## Introduction

An electrocardiogram (ECG) is not always immediately available in individuals with suspected acute coronary syndromes. Smartwatches are widespread and increasingly being used for digital health information. Apple Watch Series 4 (Apple Inc) introduced an integrated ECG tool that allows recording a single-lead ECG.^1,2,3^ This smartwatch can reliably detect atrial fibrillation and has received US Food and Drug Administration approval.^4,5^

Previous studies have explored the possibility for use of the smartwatch to record multiple ECG leads.^6^ There are also anecdotal reports of smartwatch use in patients with acute myocardial ischemia.^3,6^ However, to our knowledge, there are no studies that prospectively assessed the use of a smartwatch in a series of patients with acute coronary syndromes. Accordingly, the present study aimed to assess the feasibility and agreement of a smartwatch compared with a standard 12-lead ECG in patients with acute coronary syndromes.

## Methods

The study population included 100 individuals: 54 symptomatic patients (54%) with an ST-segment elevation myocardial infarction (STEMI), 27 symptomatic patients (27%) with a non–ST elevation myocardial infarction (NSTEMI) admitted to the coronary care unit of our division, and 19 healthy individuals (19%) as controls. The study was conducted from April 19, 2019, to January 23, 2020. The ethical committee of Magna Graecia University approved the study and all participants included gave written informed consent; participants did not receive financial compensation. This study followed the reporting guideline for case series.

**Figure 1.**
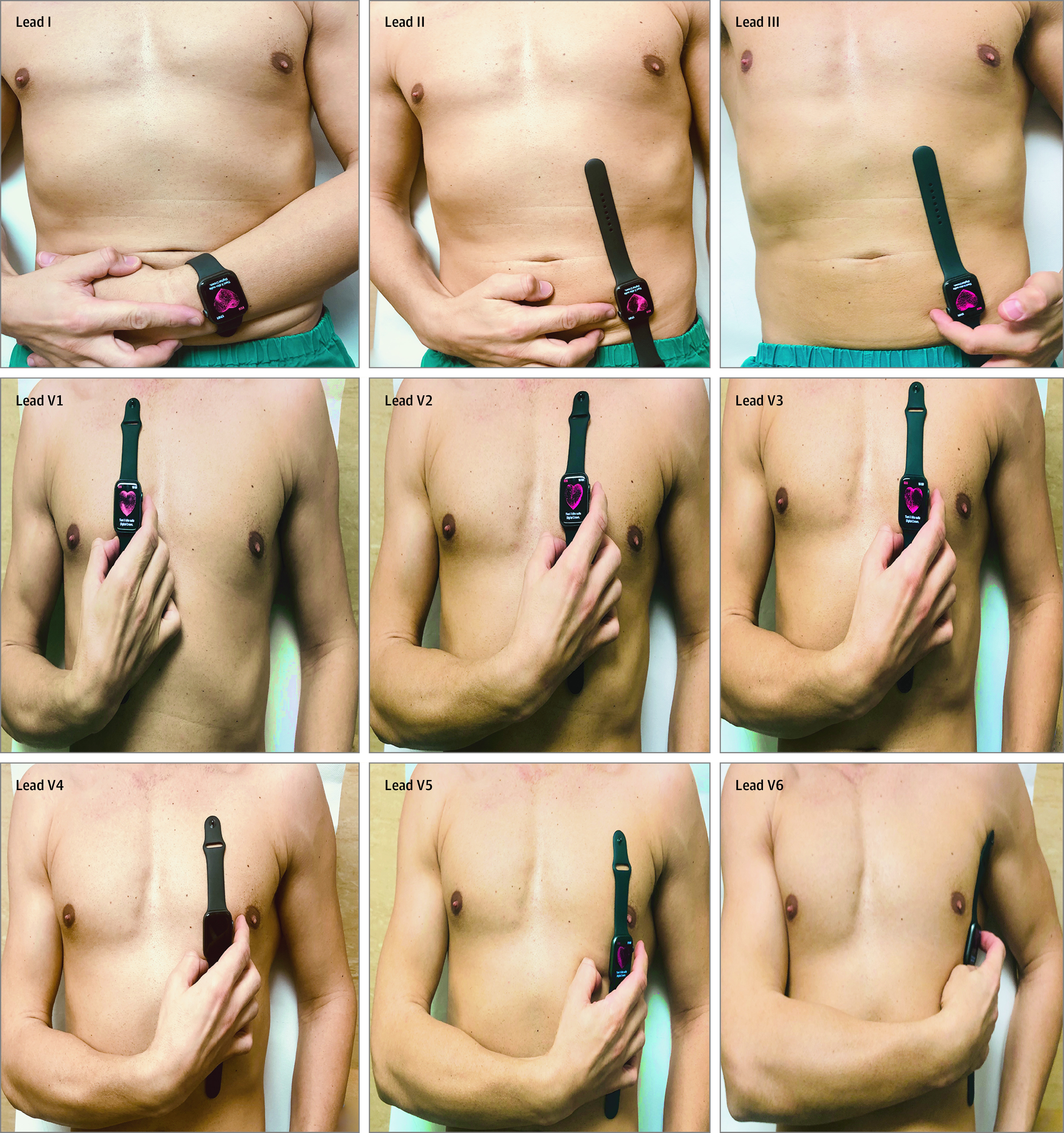
The Positions of the Smartwatch to Obtain 9-Lead Electrocardiograms (ECGs) The multiple-lead ECG with the smartwatch was obtained as follows: lead I was recorded without the removal of the smartwatch on the left wrist using the right index finger on the crown. The recording of other leads required the removal of the watch and proper placement at appropriate abdomen and chest locations. Lead II was obtained with the watch on the left lower abdomen and the right index finger on the crown, and lead III was obtained with the watch on the left lower abdomen and the left index finger on the crown. The chest leads were recorded corresponding to the location of V1 (fourth intercostal space right parasternal), V2 (fourth intercostal space left parasternal), V3 (between V2 and V4), V4 (lead at the fifth intercostal space midclavicular line), V5 (lead at the fifth intercostal space anterior axillary line), and V6 (lead at the fifth intercostal space midaxillary line).

Participants used the ECG app in the Apple Watch Series 4 smartwatch to record the ECGs. Standard 12-lead ECGs were performed (MAC 5500; GE Healthcare) with a paper speed of 25 mm/s. The attending physician of the day (ie, not the patient alone) placed the smartwatch on different body positions as shown in Figure 1. In women and in few obese individuals, the smartwatch was placed in the same positions used for the standard ECG. All recorded ECGs were digitally stored using the health application of a smartphone (iPhone Series 11 Pro; Apple Inc). All ECGs were analyzed by 2 blinded, experienced cardiologists (C.A.S. and C.I.).

### Statistical Analysis

Continuous variables are presented as mean (SD). For assessment of differences of metric outcome variables, we used paired *t* tests or Wilcoxon signed rank tests as appropriate. In the case of binary variables, we used the χ^2^ test. A *P* value <.05 was considered statistically significant. The concordance among the results of the 2 technologies was assessed using the Cohen κ coefficient. A comparison of the difference in ST-segment deviation between the 2 methods was performed using the Bland-Altman method for analysis of measurement agreement.^7^ Statistical analysis was performed using MedCalc, version 14.8 (MedCalc Software Ltd).

## Results

The study population is described in the Table. Of the 100 participants in the study, 67 were men (67%), 33 were women (33%), and mean (SD) age was 61 (16) years. The Cohen κ coefficients for the identification of normal ECG were 0.90 (95% CI, 0.78-1.00); ST-segment elevation changes, 0.88 (95% CI, 0.78- 0.97); and non–ST-segment elevation changes, 0.85 (95% CI, 0.74-0.96).

**Table.** Baseline Characteristics of the Study Population

Variable	No. (%)			*P* value
	All (n = 100)	ACS (n = 81)	CTRL (n = 19)	
Age, mean (SD), y	61 (16)	66 (10)	42 (21)	<.001
Men	67 (67)	63 (78)	6 (32)	<.001
Women	33 (33)	18 (22)	13 (68)	<.001
Hypertension	74 (74)	66 (81)	8 (42)	<.001
Diabetes	24 (24)	21 (26)	3 (16)	.35
Dyslipidemia	62 (62)	58 (72)	4 (21)	<.001
Smokers	21 (46)	21 (26)	0	<.001
Prior MI	15 (15)	15 (19)	0	<.001
Prior stroke/TIA	4 (4)	3 (4)	1 (5)	.76
Obesity	7 (7)	6 (7)	1 (5)	.74
STEMI	54 (54)	54 (67)	0	<.001
Smartwatch recording time, mean (SD), min	5.80 (0.66)	5.73 (0.73)	5.90 (0.53)	.27

Abbreviations: ACS, acute coronary syndrome; MI, myocardial infarction; STEMI, ST-segment elevation myocardial infarction; TIA, transient ischemic attack.

**Figure 2.**
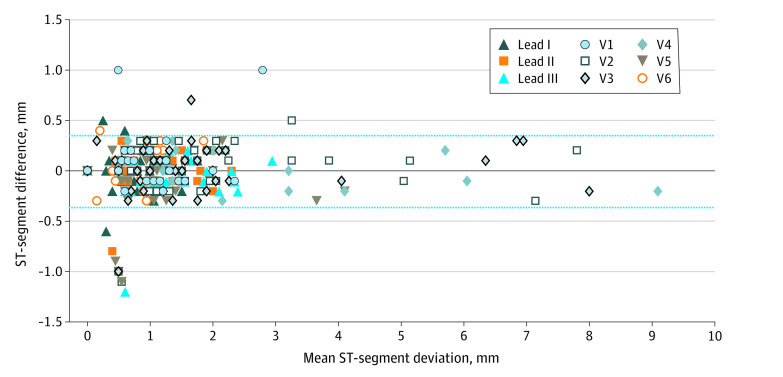
Comparison of the Amplitude of ST-Segment Deviations Between Smartwatch and Standard Electrocardiogram (ECG) Bland-Altman plot indicating the level of agreement between the smartwatch ECG and standard ECG measurement of ST-segment deviations. The black line represents the bias (mean difference), and the dashed lines represent the upper and the lower limits of agreement (bias and 1.96 SD). This difference is considered clinically nonsignificant.

Concordance was found between the smartwatch ECG and standard ECG (bias, −0.003; SD, 0.18; lower limit, −0.36; and upper limit, 0.36) using the Bland-Altman analysis. Figure 2 shows the difference in millimeters of the ST deviation between the smartwatch ECG and standard ECG plotted against the mean of the 2 readings. This difference was considered clinically nonsignificant. Furthermore, there was overall agreement for the localization of ST-segment alterations (anterior, inferior, and lateral) (Cohen κ, 0.66; 95% CI, 0.79-0.96). Representative examples of a patient with STEMI and a patient with NSTEMI are reported in the eFigure in the Supplement.

Assuming the results of standard ECGs as the reference values, STE deviation showed sensitivity of 93% (95% CI, 82%-99%) and specificity of 95% (95% CI, 85%-99%); NSTE ECG alterations were 94% (95% CI, 81%-99%) for sensitivity and 92% (95% CI, 83%-97%) for specificity. The interobserver variability analysis for 2 cardiologists (C.A.S. and C.I.) showed a Cohen κ value of 0.96 (95% CI, 0.9-1.0).

Three patients were excluded owing to low smartwatch signal quality. The first of these patients had Parkinson disease, the second patient was unable to keep their fingers on the crown owing to a previous stroke, and in a third patient, the signal was poor owing to his lack of cooperation. Three additional patients were excluded for clinical instability.

## Discussion

The major findings of the present study were that a commercially available smartwatch allowed the possibility to obtain leads I, II,III, V1, V2, V3, V4, V5, and V6, and this watch was able to detect ECG changes similar to those noted with a standard 12-lead ECG in patients with acute coronary syndromes. It has been shown that the recording of leads I to III by a smartwatch is accurate and comparable to standard ECG in healthy individuals.^2^ A recent report suggested the possibility to diagnose myocardial infarction using the smartwatch in 2 patients in whom only leads I to III were recorded with an Apple Watch.^3^ With the same smartwatch, Samol et al^6^ recorded leads V1, V2, V3, V4, and V6 (but not V5) in 2 patients with acute anterior myocardial infarction. In addition, Cobos Gil^8^ reported 2 patients (1 with STEMI and 1 with NSTEMI) in whom leads I, II, III, V1, V2, V3, V4, V5, and V6 leads were obtained with an Apple Watch.

The results of our study suggest that, in patients with ACS, in addition to detecting changes in the ST segment (Figure 2), the smartwatch was able to detect the localization of ST alterations. Although Holter monitoring has been shown to detect asymptomatic myocardial ischemia,^9^ it cannot be used as a screening tool for detecting coronary artery disease or for evaluating the severity of ischemia in individual patients.^10^

### Clinical Relevance

In patients with acute myocardial infarction, especially in a high-risk population,^2^ increased mortality was associated with treatment delays; every minute counts and 10 minutes or less is recommended from the first medical contact to recording of an ECG.^11^ The smartwatch is not designed for clinical settings such as rapid triage, emergency department, ambulance, and fieldwork. However, the possibility that, under specific circumstances (eg, when the standard ECG is not available or during pandemics^12^ or catastrophes), a smartwatch can be used to recognize ST-segment changes with multichannel ECGs that could be of clinical and social relevance in individuals with chest pain.

### Limitations

There are several limitations to the study. First, the detection of ST-segment abnormalities is not possible by simply wearing wrist smartwatch; rather, recording the ECG (except for lead I) requires removal of the watch and its placement in the appropriate chest and abdomen locations (Figure 1). Whether artificial intelligence will solve this problem has not been proven. Moreover, interpretation of the ECG that is generated by the smartwatch in PDF format must be carried out by the cardiologist because, to our knowledge, no software is available to allows interpretation of the ECG and self-diagnosis. In this regard, there is a need for the development of more easily configured and durable technology.

The lack of availability of leads aVR, aVL, and aVF using the smartwatch might reduce the sensitivity and positive predictive value for the diagnosis and localization of acute myocardial infarction. In addition, our control participants were healthy but not age- or sex-matched with the patients.

## Conclusions

The findings of this feasibility study suggest ST-segment changes on ECG shown with use of a smartwatch agree with those determined with standard ECGs. This agreement may allow the potential for earlier diagnosis of acute coronary syndromes using smartwatch technology.

## Notes

Supplement. **eFigure.** Representative Examples of ST-Segment Changes by a Smartwatch ECG.
