# Supplementary material for: Multichannel Electrocardiograms Obtained by a Smartwatch for the Diagnosis of ST-Segment Changes
Source: JAMA Cardiol. 2020 Aug 31;5(10):1176–80. doi: 10.1001/jamacardio.2020.3994 (PMC7466842; doi:10.1001/jamacardio.2020.3994)
Supplement: Supplement. — eFigure. Representative Examples of ST-Segment Changes by a Smartwatch ECG. [file jamacardiol-e203994-s001.pdf]

**Supplementary Online Content**

Spaccarotella CAM, Polimeni A, Migliarino S, et al. Multichannel electrocardiograms obtained by a smartwatch for the diagnosis of ST-segment changes. *JAMA Cardiol*. Published online August 31, 2020.
doi:10.1001/jamacardio.2020.3994

**eFigure.** Representative Examples of ST-Segment Changes by a Smartwatch ECG

This supplementary material has been provided by the authors to give readers additional information about their work.

© 2020 Spaccarotella CA et al. *JAMA Cardiology*.

SUPPLEMENTAL FIGURE 1

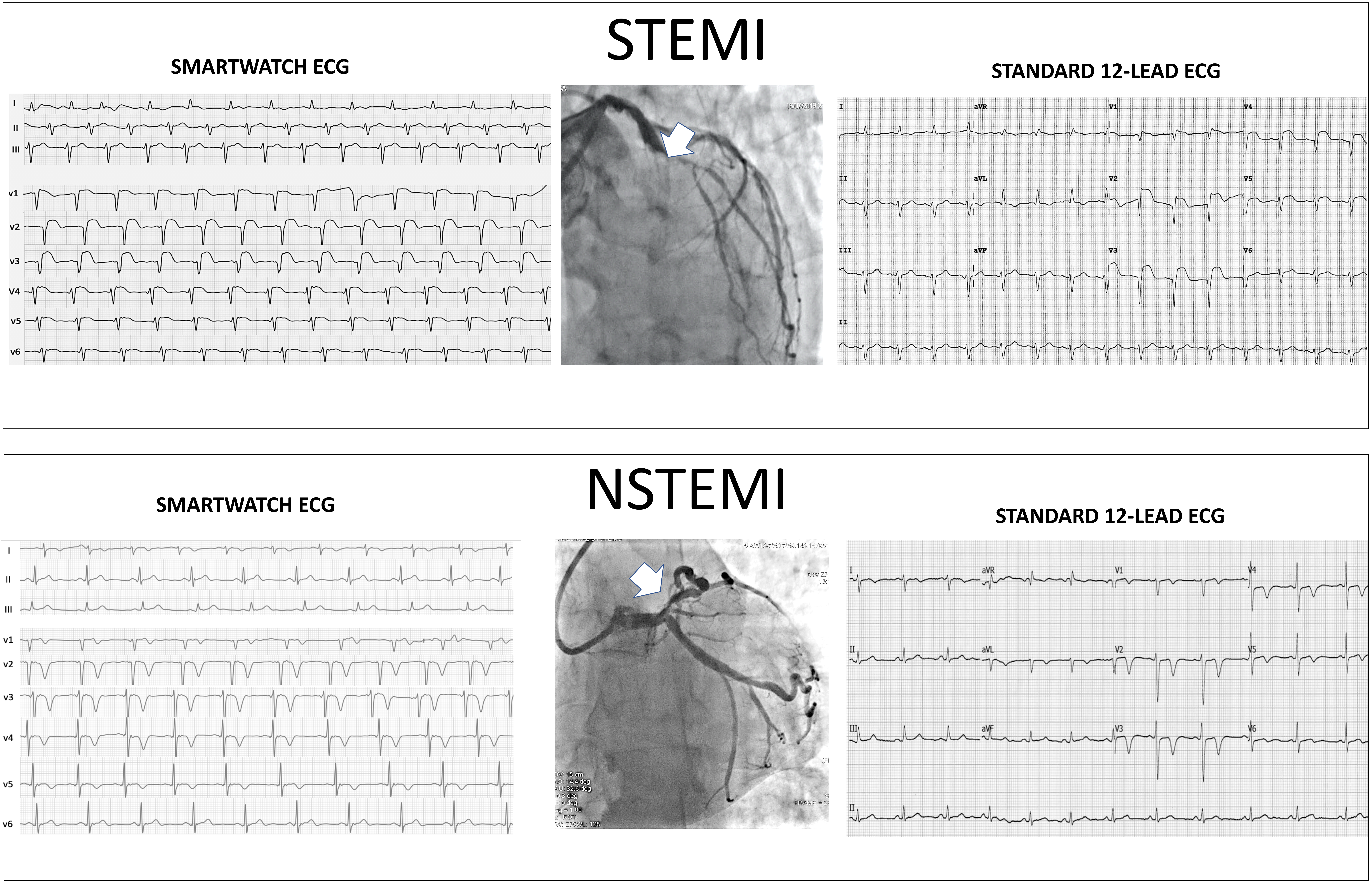


eFigure. Representative Examples of ST-Segment Changes by a Smartwatch ECG
A representative example of ST-segment elevation detected by standard electrocardiography (right panel, upper) and by a smartwatch (left panel, upper) in the same patient with an anterior myocardial infarction and total LAD occlusion (arrow, center upper panel). A representative example of the multichannel ECGs performed by the smartwatch (lower panel, left) and with standard ECG (lower panel, right) in a patient with non-ST elevation myocardial infarction

© 2020 Spaccarotella CA et al. *JAMA Cardiology*.
